# Longitudinal microbial and molecular dynamics in the cystic fibrosis lung after Elexacaftor–Tezacaftor–Ivacaftor therapy

**DOI:** 10.1186/s12931-023-02630-z

**Published:** 2023-12-16

**Authors:** Christian Martin, Douglas V. Guzior, Cely T. Gonzalez, Maxwell Okros, Jenna Mielke, Lienwil Padillo, Gabriel Querido, Marissa Gil, Ryan Thomas, Marc McClelland, Doug Conrad, Stefanie Widder, Robert A. Quinn

**Affiliations:** 1 Department of Biochemistry and Molecular Biology, Michigan State University, East Lansing, MI USA; 2 Department of Microbiology and Molecular Genetics, Michigan State University, East Lansing, MI USA; 3 Department of Medicine, University of California San Diego, La Jolla, CA USA; 4 Department of Pediatrics and Human Development, Michigan State University, East Lansing, MI USA; 5 Corewell Health, Grand Rapids, MI USA; 6 Department of Medicine, Research Division Infection Biology, Medical University of Vienna, Vienna, Austria

**Keywords:** Cystic fibrosis, Microbiome, Metabolome, Neutral models, Elexacaftor–Tezacaftor–Ivacaftor, *Pseudomonas aeruginosa*, Lung pathogens

## Abstract

**Background:**

Cystic fibrosis (CF) is a genetic disorder causing poor mucociliary clearance in the airways and subsequent respiratory infection. The recently approved triple therapy Elexacaftor–Tezacaftor–Ivacaftor (ETI) has significantly improved lung function and decreased airway infection in persons with CF (pwCF). This improvement has been shown to occur rapidly, within the first few weeks of treatment. The effects of longer term ETI therapy on lung infection dynamics, however, remain mostly unknown.

**Results:**

Here, we applied 16S rRNA gene amplicon sequencing, untargeted metabolomics, and neutral models to high-resolution, longitudinally collected sputum samples from pwCF on ETI therapy (162 samples, 7 patients) and compared to similarly collected data set from pwCF not taking ETI (630 samples, 9 patients). Because ETI reduces sputum production, samples were collected in freezers provided in the subject’s homes at least 3 months after first taking ETI, with those on ETI collecting a sample approximately weekly. The lung function (%ppFEV1) of those in our longitudinal cohort significantly improved after ETI (6.91, SD = 7.74), indicating our study cohort was responsive to ETI. The daily variation of alpha- and beta-diversity of both the microbiome and metabolome was higher for those on ETI, reflecting a more dynamic microbial community and chemical environment during treatment. Four of the seven subjects on ETI were persistently infected with *Pseudomonas* or *Burkholderia* in their sputum throughout the sampling period while the total bacterial load significantly decreased with time (R = − 0.42, p = 0.01) in only one subject. The microbiome and metabolome dynamics on ETI were personalized, where some subjects had a progressive change with time on therapy, whereas others had no association with time on treatment. To further classify the augmented variance of the CF microbiome under therapy, we fit the microbiome data to a Hubbell neutral dynamics model in a patient-stratified manner and found that the subjects on ETI had better fit to a neutral model.

**Conclusion:**

This study shows that the longitudinal microbiology and chemistry in airway secretions from subjects on ETI has become more dynamic and neutral and that after the initial improvement in lung function, many are still persistently infected with CF pathogens.

**Supplementary Information:**

The online version contains supplementary material available at 10.1186/s12931-023-02630-z.

## Introduction

Cystic fibrosis (CF) is caused by homozygous recessive mutations in the cystic fibrosis transmembrane conductance regulator (*CFTR*) gene [1]. This gene encodes the CFTR protein, whose role is to balance the normal traffic of chloride ions and water in the airway surfaces. Additionally, dysfunction of CFTR proteins leads to an osmotic imbalance that results in desiccated mucous secretions and respiratory infection by opportunistic pathogens (particularly *Pseudomonas aeruginosa*, *Staphylococcus aureus,* and others) [2]. Antibiotics, anti-inflammatory agents, mucolytics, and other pharmaceutical approaches are available to treat the symptoms and bacterial infections of CF, all showing some benefit to patient symptoms [3, 4]. In the last decade, substantial improvements in the lung function of people with CF (pwCF) have been achieved by targeting CFTR defects with small-molecule protein correctors and potentiators. Most recently, the triple therapy Elexacaftor–Tezacaftor–Ivacaftor (ETI, TRIKAFTA^®^) has been approved to treat those with at least one copy of the common F508del mutation and preliminary results show remarkable efficacy for improving symptoms of CF and lung function [5–7]. A recent study showed that the improvement is rapid, with increases in lung function and decreases in sputum pathogen load occurring within the first month followed by a new steady state where infectious load and lung function improvement stay relatively stable through 6 months after starting ETI [7]. It is of paramount importance to understand if lung infection and biochemical profiles continue to change with time on ETI in a predictable manner as studies of previously approved CFTR modulators showed a resurgences of pathogen infection after the period of initial improvement [8, 9]. Furthermore, information on how ETI is affecting microbial and chemical dynamics in the airways on more high-resolution longitudinal timeframes is completely unknown.

Multi-omics studies, including metagenomic, metabolomic, transcriptomic, and many others, are a powerful integrated approach to monitoring changes in complex microbial and host systems. These methods have been extensively applied to study CF lung infections and the immune system in cross-sectional studies [10–14], revealing that the CF lung microbiome presents as an extreme dysbiosis, where the respiratory tract is infected with a high load of opportunistic pathogens and airway commensals that adapt and evolve with the patient over their lifetime [11]. The metabolome of the CF lung has been less well-studied but is known to contain high levels of mucin, DNA, amino acids, microbial virulence factors, and pharmaceuticals [15–17]. A recent study linked peptides and amino acids in sputum to lung function decline and small molecule virulence factors from the bacterial pathogen *P. aeruginosa* are readily detected in airway secretions of pwCF [10]. Importantly, the concentration of amino acids and peptides were shown to decrease in sputum upon ETI administration [12]. Applying these powerful techniques to high-resolution longitudinal study designs provides a unique view of the microbial and molecular dynamics of complex microbial systems, such as the CF lung. A better understanding of the effects of ETI on CF lung disease through time could help understand how the drug is providing such strong symptom relief and improvement of lung function in pwCF.

Here, we paired 16S rRNA gene sequencing, quantitative polymerase chain reaction (qPCR), untargeted metabolomics and neutral models to longitudinally collected sputum samples from pwCF taking ETI. We were particularly interested in capturing microbial dynamics that were occurring after the previously reported rapid reduction in infectious load after 1 month of therapy [8]. For a control group, we compared our findings to a similarly collected dataset of sputum from subjects not taking ETI, some of which was previously published [18]. The data reveals that the lung microbiome and metabolome of subjects on ETI are more dynamic, changing more rapidly through time, though overall, sputum produced by subjects on this new therapy still have significant pathogen loads and omics signatures from the era of CF prior to ETI approval.

## Methods

### Sampling collection and clinical information of study subjects

**Fig. 1:**
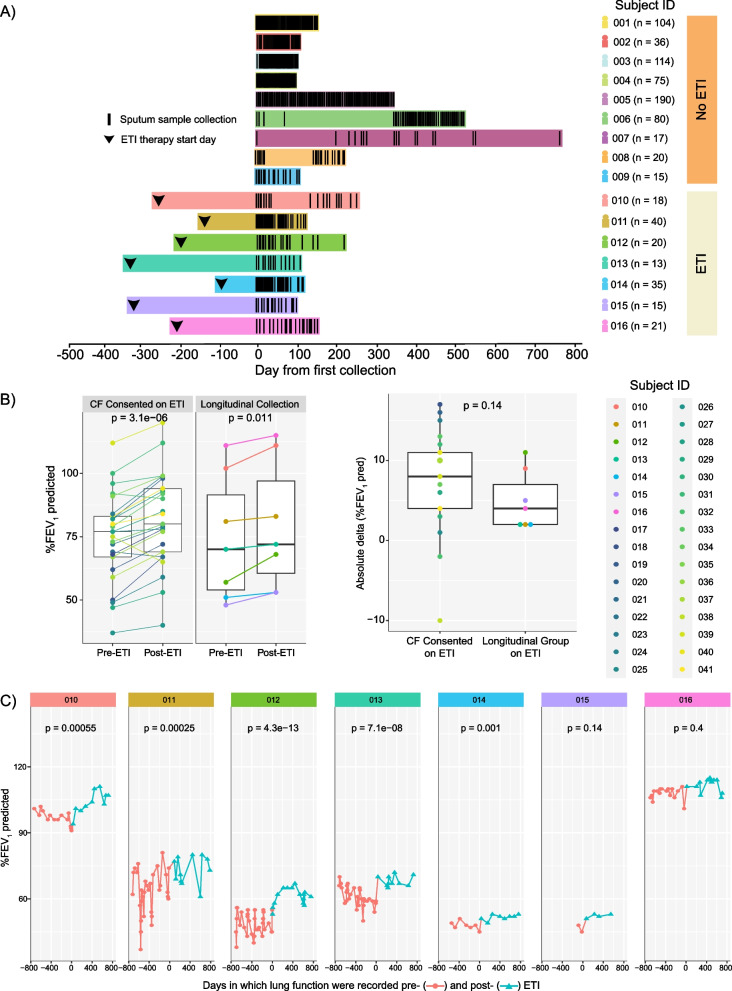
Sampling collection data through time and lung function response to ETI treatment among participants. **A** Schematic of longitudinal samples collected for this study. Each black line represents a sample collection day. Black arrowheads represent the day in which subjects started on ETI treatment. **B** Lung function variation for highest ppFEV1% predicted pre- and post-ETI treatment per subject on each group and the absolute delta variation in the change in ppFEV1% predicted per subject post-ETI treatment within each group. The significance was determined through the DM t-test and Welch’s t-test respectively. **C** Lung function as ppFEV1% predicted of individual subjects through time (days) before and after ETI. Colors represent the treatment status (pre- and post-ETI) in which lung function was recorded and its corresponding significance (T-test) between the two different periods

Prior to sample collection, explicit written consent was obtained from each participating subject. Thus, sputum samples (n = 162) from pwCF on ETI therapy (n = 7) were longitudinally collected at home and compared to a similarly collected (n = 578), previously published data set of CF subjects (n = 6) [18] along with 52 newly collected samples provided by 3 additional subjects not taking ETI (Fig. 1A). Newly studied subjects were asked to collect a sputum sample weekly in a 50 mL conical tube and place it in a frost-free − 20 °C freezer provided in their own home by the study team. Sputum sample collection was at the discretion of the subjects, such that if a sample could not be produced, it was simply not collected. Because of the ease in producing sputum prior to ETI approval, subjects not taking ETI collected more frequently (Fig. 1A). Clinical and demographic information such as lung function (ppFEV1, FVC), body mass index (BMI), and gender were recorded among other parameters of interest (Table 1). Inclusion criteria for the study included: diagnosis of cystic fibrosis, > 18 years of age, ability to produce sputum at home and consent to placement of a − 20 °C freezer without an automatic defrost feature in their home for collection. The exclusion criteria for this study were the inability to spontaneously produce sputum or tolerate the collection procedure. Because ETI reduced sputum production for many pwCF, but our study cohort was able to produce sputum at home, we compared the lung function improvement of our longitudinal cohort with other consented subjects from the University of California San Diego (UCSD) adult CF clinic (n = 26) to determine if they had a varied response to ETI. The best ppFEV1%-predicted within a year pre- and post-ETI was used to compare clinical response between the longitudinal cohort studied here and the others. Ethical approval for the collections at the UCSD adult CF clinic was obtained from the UCSD Human Research Protections Program Institutional Review Board under protocol #160078.

**Table 1:** Clinical, demographic, and sample characteristics of pwCF (n = 16) on/off ETI therapy (n = 7/9)

Subject ID	ETI	Days on ETI	Period (days) between the first sample and ETI	Samples collected	BMI	Gender	ppFEV1 PP (%)	FVC PP (%)	Pathogen cultures (Sputum)
001	No	–		104	18.9	F	54.4	73.8	–
002	No	–		36	27.5	M	54.8	73.1	–
003	No	–		114	23.0	M	38.7	48.3	Pa, Sa
004	No	–		75	20.6	F	52.9	63.4	Pa, Ac
005	No	–		190	29.0	M	69.6	91.1	Steno
006	No	–		90	18.5	F	52.0	78.4	–
007	No	–		17	30.2	F	50.0	60.0	Pa, Sa, Ac, Strep
008	No	–		20	21.5	F	52.0	70.0	Pa, Sa
009	No	–		15	22.1	M	40.0	66.0	Pa
010	Yes	537	277	18	27.3	F	111.0	114.0	Pa, Sa
011	Yes	286	163	40	27.9	M	79.0	100.0	Pa, Sa, Steno, Strep
012	Yes	454	226	20	20.2	F	67.0	84.0	Pa, Sa, Strep
013	Yes	453	345	13	23.3	F	72.0	75.0	Pa, Sa, Bc,
014	Yes	227	103	35	20.7	F	53.0	–	Bc
015	Yes	426	326	15	22.4	F	52.0	70.0	Pa
016	Yes	377	210	21	23.2	F	101.0	107.0	Pa, Sa

Subjects id’s information (001-006) was obtained from [18]. ETI treatment status as well as the time (days) on treatment since started until the last sample collection, body mass index (BMI), gender, highest predicted lung function (ppFEV1 and FVC), and pathogen cultures within sputum are presented. Pathogen results are abbreviated as follows: *Pseudomonas aeruginosa* (*Pa*), *Staphylococcus aureus* (*Sa*), *Burkholderia cepacia* (*Bc*), *Achromobacter* sp. (*Ac*), *Stenotrophomonas* sp. (*Steno*) and *Streptococcus* sp. (*Strep*). The subject’s IDs were decoded as serial numbers to keep the anonymity and privacy of participants.

### DNA extraction and 16S rRNA amplicon sequencing

DNA extraction from the newly collected sputum was performed using the Quick-DNA Miniprep Plus Kit (Zymo® Research) following the standard protocol for biological fluids and cells. The bacterial 16S rRNA V4 amplicon sequencing was conducted with primers 515F (5′-GTGCCAGCMGCCGCGGTAA-3′) and 806R (5′- GGACTACHVGGGTWTCTAAT-3′) on an Illumina® MiSeq® at the Michigan State University Sequencing Core following the protocol described in [19]. The raw sequences were processed, trimmed at 150 base pairs, rarefied to 11,030 (sampling depth), and demultiplexed using QIITA (qiita.ucsd.edu) [20], which applies QIIME2-based algorithms [21], and quality filtered to generate amplicon sequence variants (ASVs) through the Deblur method [22]. Taxonomy was assigned using q2-feature-classifier against the 99% GreenGenes 16S rRNA reference database (version 13-8) [23]. As negative control, we used samples containing no DNA, and as positive control we used a mock community consisting of *P. aeruginosa*, *S. aureus*, *Streptococcus gordonii*, *Achromobacter xylosoxidans*, *Rothia mucilaginosa*, *Stenotrophomonas maltophilia*, *Granulicatella elegans*, *Veillonella atypica*, and *Prevotella melaninogenica*) to monitor both the potential contamination and the sequencing consistency. The raw sequences are deposited in the European Bioinformatic Institute (PRJEB70440, ERP155373) and can also be accessed through the Qiita study 14652. This microbiome data was integrated with the data from the previously published longitudinal collections already available in Qiita [18]. The PCR and amplicon sequencing methods were identical between the newly generated ETI cohort and those previously published [18], however, the DNA extraction kit used for the previously published data was the Qiagen Powersoil® kit. The data from both cohorts was exported and processed with the phyloseq package in R [24].

The extracted DNA from sputum samples of subjects on ETI was also used to calculate the total bacterial load through qPCR. Thus, two universal primers 515F and 806R were used in qPCR to amplify the 16S rRNA gene [25, 26]. The reaction was performed in duplicates of 12.5 μL using power SYBR Green PCR master mix (Applied Biosystems). The reactions were run on QuantStudio3 thermocycler (Thermo). The standard curves of a diluted culture of *Pseudomonas aeruginosa* DNA with a known CFU/mL extracted with the same procedure were used to determine an estimate of the total rRNA gene copies per mL of media after adjusting for the four rRNA gene copies in the *P. aeruginosa* genome. The average of log values (rRNA gene copies) obtained from these technical replicates were used in subsequent analyses.

### Organic extraction, liquid chromatography-tandem mass spectrometry (LC–MS/MS), and metabolomics data processing

Organic metabolite extraction was performed by adding twice the sample volume of chilled 100% methanol, vortex briefly, and incubating at room temperature for 2 h. Samples were then centrifuged at 3000×*g* for 10 min to pellet precipitated protein and the supernatant was collected. Extracts were analyzed on a Thermo Q-Exactive® Hybrid Quadrupole-Orbitrap mass spectrometer coupled to a Vanquish® ultra-high-performance liquid chromatography system. Briefly, sputum metabolites were separated on an Acquity C18-Reverse phase column (Waters) with a 12 min chromatography run using 0.1% formic acid in acetonitrile (channel A) and Mili-Q water (channel B) gradient (98:2 to 2:98). The injection volume was 10 μL, the flow rate was 0.40 mL/min, and the column temperature was 60 °C. Full MS^1^ survey scans and MS^2^ mass spectra for five precursor ions per survey scan were collected using electrospray ionization with a scan range set from *m*/*z* 100 to 1500 for the full MS mode (1–10 min of run) [12, 18]. Raw files were converted to.mzXML format and then processed with MZmine software (versions 2 and 3, respectively), Global Natural Products Social Molecular Networking online platform (GNPS), and SIRIUS (version 5.7) [27–29]. The MZmine 3 settings for the new group of samples are available in supplementary methods. The resulting GNPS jobs (data link to https://gnps.ucsd.edu/ProteoSAFe/status.jsp?task=ee0758be77b241a3957d1424487884f6) and feature quantification tables were then used for statistical and machine-learning analyses. The metabolome of the sputum samples collected from pwCF on ETI was independently analyzed using CANOPUS through SIRIUS to longitudinally determine the in silico chemical classifications of the metabolites from pwCF on ETI [30]. The previous longitudinal sputum metabolome dataset [18] had some methodological differences with this one, including the extraction approach (ethyl acetate and methanol) and the use of a Quadrupole Time of Flight (qTOF) mass spectrometer. However, comparisons between the two datasets were only done after the calculation of variation within an individual to limit methodological confounders between the two batches.

### Statistical analysis

We first tested the normality of the various data type distributions including using a Shapiro–Wilk (SW) test to determine the appropriate statistical methods [31]. If normal, paired dependent means t-tests (DM t-test) were conducted to evaluate the pre- and post-ETI paired measures and Welch’s t-tests were used to evaluate differences between means that were not dependent. If normality was not identified, Wilcoxon signed-rank tests were used to compare measurements with and without ETI. The microbiome and the metabolome data were uploaded to QIITA (qiita.ucsd.edu) [20] as.biom tables for calculating the alpha- and beta-diversities. Alpha-diversity was calculated using the Shannon index while beta-diversity used the weighted UniFrac (microbiome) and Bray–Curtis dissimilarity distance metrics (metabolome). Data from the previously published longitudinal sputum collection of pwCF not on ETI (n = 6,) was integrated with data generated anew for this study. To minimize batch effects between the two collections, alpha- and beta-diversity changes were only calculated as change per day within each subject and then compared across the ETI and non-ETI groups. This compares the degree of variation within each subject for the microbiome and metabolome data which is less likely to be affected by any differences in the two data batches. All other comparisons in the study were only done within the ETI group through time.

To identify associations between the multi-omics data and time on ETI, random forest (RF) [32] regression analysis was performed for each subject’s microbiome and metabolome data. Linear regression analysis was used to determine the significance of the correlation between the RF predicted and the actual observed time since ETI. Plots were performed through the packages ggplot2 (version 3.4.3), phyloseq (version 1.40.0), vegan (version 2.6.4), ggpubr (version 0.6.0), patchwork (version 1.1.3) in RStudio (version 4.2.1) [33–37].

### Data neutral modeling

To compare CF microbiome dynamics with and without therapy, we fitted rarified 16S data to a simplified neutral community model for prokaryotes [38], developed as maximum likelihood model [39]. We implemented a sample stratification scheme to correct for subject-specific sampling frequencies, specified as follows: Given a fixed time-interval of 100 days, 12 samples were randomly selected without replacement and aggregated as a subset for model fit. The subsets were collected in a sliding time window along the patient trajectory. The procedure was repeated 100 times, all subsets were fitted, and mean values of model fits were reported for the respective time intervals. Subsampling and model fit was implemented in R using the function *sncm.fit()* available from [38]. Of note, this stochastic model implementation minimizes the log-likelihood (LL) of the loss function, i.e., lower LL reflects a better fit. Fit statistics were assessed in a subject-specific manner, goodness of fit was estimated using Akaike information criterion (AIC) and a generalized R^2^, whereas model error was assessed employing residual mean square error (RMSE). Group-wise value comparisons were performed with non-parametric using Wilcoxon rank-sum tests and plotted using ggplot2 [33].

## Results

### Sample collection and clinical design

The objectives of this study were to determine if the microbiome and the metabolome of sputum from pwCF on ETI therapy (n = 7) changed through time within the first 300 days of starting therapy, with a particular focus on the time period after 1 month on treatment [8], and if these dynamics were different from those not on ETI. As a control group, our longitudinal data was compared to sputum samples similarly collected in home freezers from those not taking ETI (n = 9). Six of the non-ETI subject’s samples and data were previously published in a longitudinal study of microbial and metabolite dynamics of CF [18], and three additional subject’s collections were added for this study (Fig. 1A). There is no overlap of subjects between each group. Clinical parameters, medical treatments, and patient demographic information are presented in Table 1 and Additional file 1: Table S1. All subjects in both groups were asked to produce sputum samples ad libitum at home and store the samples in home freezers provided by the study team. The ETI group was asked to provide a sample at least weekly, but this was not always possible due to the reduction in sputum production in this group, and some subjects collected more often. Most of the collections were performed during the COVID-19 pandemic, which may have an unknown impact on our results due to social distancing or other factors, but the home study design facilitated the collection of samples for this study when routine clinical visits were greatly reduced. However, challenges with delivering freezers and consenting patients during the pandemic were encountered, therefore, not all subjects began sample collection at the same time after taking ETI. The average collection period for subjects on ETI was 267 days (SD = 106), the average start of collection days after taking ETI was 236 (SD = 87) while the average number of samples collected from subjects on/off ETI is 23.14 (SD = 10.28) and 73.44 (SD = 58.42), respectively.

Because the effects of ETI therapy from clinical trials and early clinical observation was a reduction in sputum production, we first aimed to determine if our sputum-producing group of ETI volunteers (n = 7) had a different clinical response to treatment measured by the percent predicted forced expiratory volume in 1 s (ppFEV1), than other consented group of pwCF taking ETI in the same study clinic. We compared the highest ppFEV1 predicted for each subject within a year pre- and post-ETI treatment and found a significant improvement post-treatment in both the CF-consented population (DM t-test, p = 3.1E−06) and our longitudinal sputum-producing group (DM t-test, p = 0.011) (Fig. 1B). Comparing absolute ΔppFEV1 improvement between the two populations was not significantly different (Welch’s t-test, p = 0.14), indicating that the longitudinal study subjects had similar responses to ETI as the clinic’s population, though their improvement trended lower (Fig. 1B). We also evaluated the lung function of the longitudinal subjects since starting ETI and found that 5 subjects (010, 011, 012, 013 and 014) displayed significant gain in the ppFEV1 during the collection period (Fig. 1C).

### Microbiome and metabolome diversity dynamics with and without ETI therapy

**Fig. 2:**
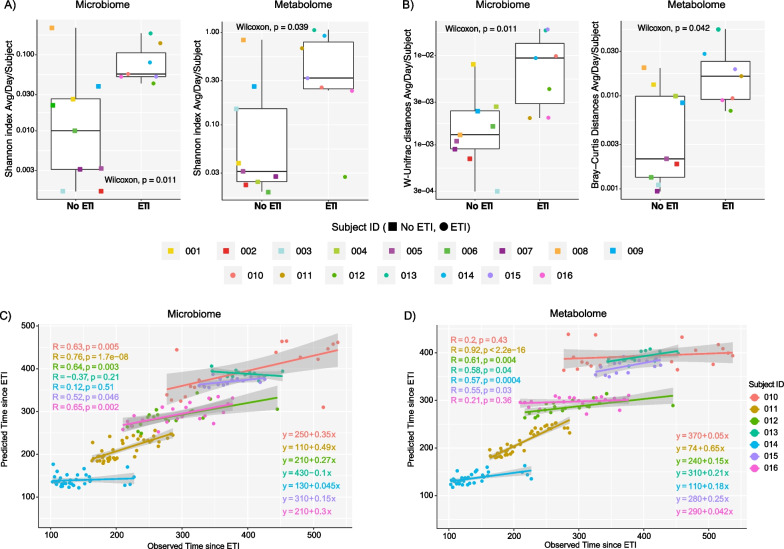
The average microbiome and metabolome diversity of subjects in this study. **A** Average change in the alpha-diversity (Shannon index) of lung microbiome and metabolome of pwCF off/on ETI treatment per day. Square shapes represent individuals who did not take ETI, while circular shapes are associated with those who did take ETI. **B** Average of the beta-diversity of the lung microbiome and metabolome of pwCF before and after ETI per day. Weighted UniFrac distance was used for microbiome data and Bray–Curtis dissimilarity for metabolome data. P-values obtained from the Wilcoxon test are displayed. Scatterplots of the predicted vs. observed **C** microbiome and **D** metabolome association with the time in days since pwCF started on ETI treatment. This data was obtained from RF regression analysis of each subject as well as their Pearson correlation tests

We measured the microbiome and metabolome alpha- and beta-diversity change per day from the sputum samples and compared those that were off ETI to those that were on treatment. Here, we found that the degree of daily increase in the ΔShannon index was higher for those on ETI in both the microbiome and metabolome (Wilcoxon test, p = 0.011 and p = 0.039, respectively). Calculation of the beta-diversity change normalized for the time between samples (ΔUniFrac for microbiome or ΔBray-Curtis for metabolome) showed that both the microbiome and metabolome of those on ETI were also changing more rapidly (Wilcoxon test, p = 0.011 and p = 0.042, respectively) (Fig. 2A, B). This data supports that the microbial community and chemical constituents of sputum were more dynamic in those taking ETI compared to our control subjects.

We then used a machine learning approach to determine if these changes had a linear association with time since ETI which would support that the data was progressively changing predictably while on therapy. Random forest (RF) regression analysis was performed by subject to determine if the algorithm could predict the time since starting the drug for each sample based on the omics data (Additional file 1: Table S2). We found that data from 5/7 subjects on ETI had a significant linear relationship in both their microbiome (010, 011, 012, 015, 016) and the metabolome (011, 012, 013, 014, 015) with time since treatment started. This indicates that these subjects have a progressively changing microbiome and metabolome since taking the drug. However, two subjects showed no linear association with time indicating that their microbiome and metabolome dynamics were more static during the study period (Fig. 2C, D).

### CF pathogen dynamics in sputum of subjects on ETI

**Fig. 3:**
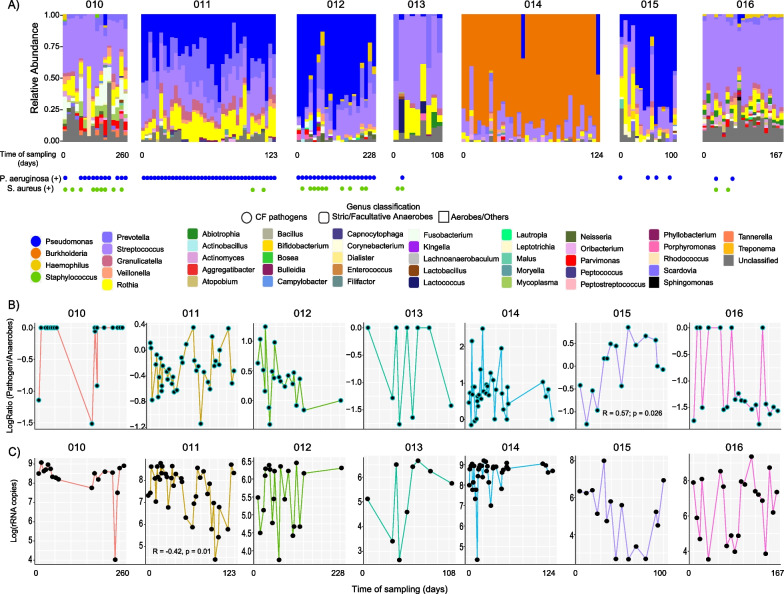
Microbiome dynamics of subjects on ETI therapy through time of sampling. **A** Bar plots representing the microbiome’s relative abundance of longitudinally collected sputum samples from seven subjects on ETI. The sample collection time point (beginning and end of days) per subject is displayed, while gaps across sampling are not shown. The results of bacterial cultures in the clinics are shown for *P. aeruginosa* and *S. aureus*. The genus level and its classification as a CF classic pathogen as well as their oxygen tolerance are also presented. **B** Line-dot plots represent the pathogen:anaerobe log-ratios and **C** log rRNA gene copies in the sputum of each subject on ETI treatment collected through time.

The genera resembling classic CF pathogens, including *Pseudomonas*, *Burkholderia*, and *Staphylococcus*, were identified in the microbiome of those on ETI as well as oral facultative and/or strict anaerobes such as *Streptococcus*, *Prevotella,* and *Veillonella*. We referenced the clinical culture record during the time of sample collection and found that our sputum-producing subjects on ETI had positive cultures of *P. aeruginosa* (6/7 subjects) and *S. aureus* (5/7 subjects) at different time points during the treatment period (Fig. 3A, Table 1). We tested whether the relative abundance of these pathogens was decreasing with time on ETI therapy within each subject. To account for the compositional nature of the microbiome data and the different pathogens in each subject, we binned the organisms into classic ‘pathogens’ or ‘anaerobes’ [18] and compared the log-ratio of pathogens/anaerobes through time on ETI. We did not find significant differences in the pathogen/anaerobe log-ratio within subjects on ETI over time except for subject 015, which saw an increase in this ratio (R = 0.57, p = 0.026) (Fig. 3B). Additionally, the total bacterial load (measured by the rRNA gene copy number) did not change significantly across all subjects on ETI through time, except subject 011 displayed a significant longitudinal decrease (R = − 0.42, p = 0.01) (Fig. 3C). We then investigated whether the presence of antibiotics in the subject’s sputum could affect the microbiome profiles through time on ETI. Because these samples were collected at home, the clinical record of when the drugs were taken was not available, however, the LC–MS/MS mass spectrometry data detected antibiotics directly in the paired samples with microbiome data including azithromycin, aztreonam, doxycycline, ciprofloxacin, trimethoprim, levofloxacin, and sulfamethoxazole (Additional file 1: Fig. S1). This data allowed for a comparison of antibiotic abundance in sputum with the microbiome alpha-diversity (Shannon index) in the same samples, which showed that azithromycin and aztreonam abundance displayed a weak correlation (positive and negative, respectively) with the alpha-diversity (Additional file 1: Fig. S2a). However, not all subjects had the same antibiotics detected, azithromycin (3/7) and aztreonam (4/7), indicating the effect may have been driven by individual microbiome differences (Additional file 1: Fig. S2b). Overall, this data demonstrates that some subjects on ETI (4 of the 7 studied here) still have pathogens in their sputum that persisted until the end of the sample collection period.

## Metabolome changes in subjects on ETI

**Fig. 4:**
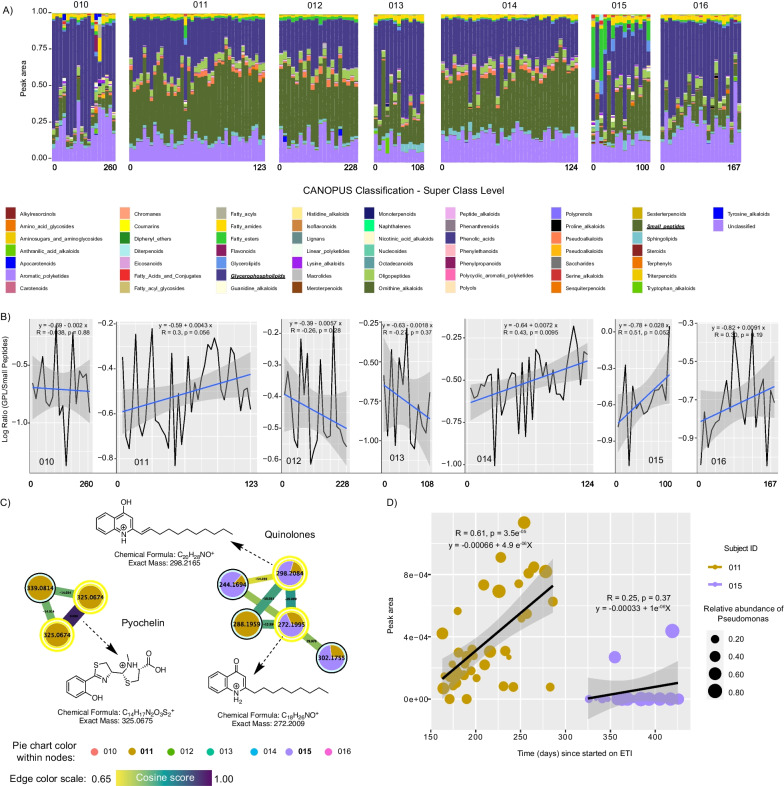
Metabolome dynamics of subjects on ETI therapy through time of sampling. **A** Bar plots representing the metabolome’s normalized peak area of longitudinally collected sputum samples from seven subjects on ETI. The sample collection time point (beginning and end of days) per subject is displayed, while gaps across sampling are not shown. **B** Line plots represent the GLP:small peptide log-ratios. **C** Molecular networking displaying *Pseudomonas*-like molecules from sputum samples of subjects on ETI. Nodes in yellow denote those molecular features annotated by GNPS. **D** Scatter plot representing the molecular dynamics between selected *Pseudomonas* metabolites, the time in days since subjects 011 and 015 started on ETI, while the size represents the relative abundance of *Pseudomonas* at the time of sampling over time

We used CANOPUS molecular family assignments to determine if different molecular families were changing across the cohort on ETI and RF variable importance plots to identify specific metabolites across the study that were changing with time. We found that the chemical composition of the sputum from the overall subjects on ETI was mainly composed of glycerophospholipids (GPLs) and small peptides (Fig. 4A). We therefore averaged the abundance of all GPLs and small peptides and compared their compositional log-ratio change with time. These log-ratios revealed a positive relationship with time on ETI in 4/7 subjects with one reaching statistical significance of the linear regression at an alpha-level of 0.05 and two others nearing significance (p = 0.052 and p = 0.056; Fig. 4B). In addition, RF analysis on molecular families changing with time (64.27% variance explained by time on ETI) revealed macrolides (Azithromycin) and amino acids had the strongest association with time on ETI (Additional file 1: Fig. S3). Due to the personalization within the metabolome, there were no individual metabolites universally changing with time on ETI across subjects.

Because of the importance of *P. aeruginosa* to CF and our ability to detect its specialized metabolites in our metabolomic data, we explored the presence and dynamics of its various small molecule virulence factors in subjects taking ETI. By searching our sputum metabolomics data against the GNPS mass spectral libraries based on their MS/MS patterns, we identified pyochelin, 2-nonylquinolin-4(1*H*)-one (NHQ) and 2-(undec-1-en-1yl)quinoline-4-ol. These molecules were detected only in subjects 011 and 015 (Fig. 4C), with only subject 011 showing a significantly positive correlation with the time on ETI (R = 0.61; p = 3.5E−05, Fig. 4D; and Additional file 1: Fig. S4), however, the production of *Pseudomonas* metabolites in subject 011 did not exhibit a discernible pattern with the changing abundance of *Pseudomonas* over time.

## Microbiome dynamics become more neutral after ETI therapy

**Fig. 5:**
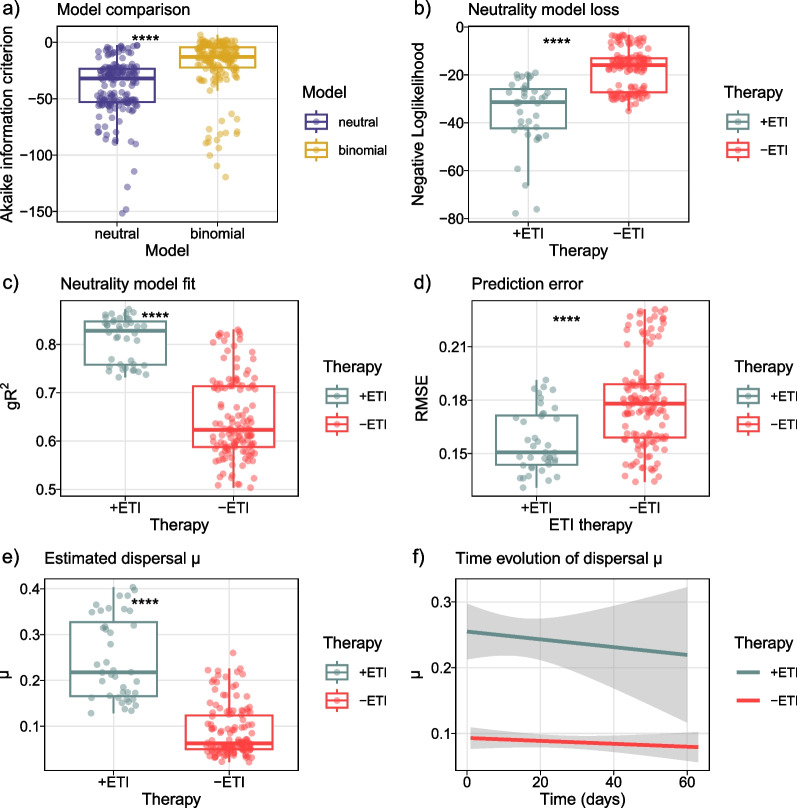
Modulator therapy is associated with increased community neutrality and immigration. **A** Model comparison between a simplified neutrality model with dispersal limitation and a stochastic binomial distribution. **B**–**D** Fitting statistics of stratified 16S data to neutrality model. **E** Estimated dispersal in communities with and without modulator therapy. **F** Time evolution of predicted dispersal in communities with and without modulator therapy, 0.95 confidence interval of regression is plotted

It has been reported that the healthy lung microbiota displayed neutral community dynamics, i.e., microbial abundances were explained by immigration from adjacent body sites and local replacement [39, 40]. This raised the question whether the observed variability under ETI treatment could be caused by changed dispersal limitations for bacteria immigrating to the lung microenvironment. To investigate this, we implemented a simplified neutrality model in parallel with a stochastic binomial model and compared fits using Akaike information criterion (AIC, Fig. 5A) [38]. We found that a simplified neutral model reflected microbial abundances better than a stochastic distribution without dispersal (Wilcoxon, p < 2e−16) and the model predicted increased immigration (Wilcoxon, p < 2E−16, Fig. 5E). However, a linear mixed model relating immigration and therapy duration correcting for subjects as random effects estimated that immigration rates decreased with treatment duration (LMM, k = − 7.8E−4, p = 7.9E−2, Fig. 5F). This may indicate that the original increase of community turnover after therapy start can reduce with time.

## Discussion

This study describes the multi-omic data changes in high-resolution longitudinally collected sputum samples from pwCF taking the highly effective CFTR modulator therapy ETI. ETI has resulted in significant improvement in the symptoms of pwCF since its approval in 2019 by the U.S. Food and Drug Administration (FDA), and now other agencies worldwide. Recent literature shows that ETI therapy is also reducing the load of opportunistic pathogens in the airways and sputum, and importantly, this reduction occurs rapidly (1 month) with a period of stasis and persistence of infection in some subjects up to 6 months on therapy [8, 41]. Similarly, lung function improvement occurs rapidly and holds, so far as can be determined from the current literature [9, 42, 43]. This contrasts with studies of prior CFTR modulators, that showed rapid improvement, but then a return of infection and lung function decline [44–47]. Importantly, this longitudinal study included sputum samples collected after the initial period of rapid change in lung microbiome and lung function from ETI therapy during an apparent period of more relative stasis [8]. The high-resolution longitudinal data was analyzed to determine if there was a continued progressive change during this period and if it indicated infection improvement. Though the number of subjects sampled was small, the sample size within individuals was high, providing a detailed view into the changing airway microbiome and its associated metabolome during ETI therapy. Our principal findings are that the lung microbiome and metabolome were more dynamic in those taking ETI and the microbiome dynamics fit better to a neutral model. We also found that some subjects still have significant pathogen loads in their sputum despite an apparent improvement in lung function.

Prior studies have noted that the sputum production drastically decreased in pwCF on ETI, including one reporting that approximately 62% either completely ceased and/or significantly reduced their sputum production within a median period of 4.3 months (ranging from 3.0 to 5.6 months) [48] and another reporting similar trends (67%) after one year of treatment [49]. Because of this beneficial therapeutic effect, we set out to determine whether subjects in this study readily producing sputum at home were somehow unique in their response to ETI or were ‘non-responders’. We tested this by comparing the lung function improvements in this at-home study group with other consented subjects from the same clinical population and found that the longitudinal cohort did improve on ETI and this was not significantly different than others. However, the mean improvement before and after therapy was lower than that population, indicating the subject’s producing sputum at home may have had a slightly reduced response. It is therefore notable that these subjects still had significant pathogen loads in their sputum, with little change in their relative abundance or total bacterial load over time, despite their improvement in lung function. The significant pathogen loads in sputum microbiomes reported here are consistent with a study in which the sputum of 236 pwCF were sampled for 6 months after ETI treatment observing pathogen persistence in many subjects from bacterial cultures, PCR, and DNA sequencing [8]. Collectively, the growing literature on microbiome changes in pwCF on ETI supports the notion that structural lung damage susceptible to infection may persist [8, 9, 42], leading to reservoirs for bacterial pathogens to continue to damage the airways. This finding argues for the importance of continued microbiological monitoring in subjects on ETI despite the improvement in their overall health.

The results reported here also show that a progressively changing microbiome and metabolome is occurring in those on CF within the first year of therapy, though not in all subjects. This may indicate personalization and variation in the longer-term response to ETI, with some subject’s infections becoming relatively static, while others continue to change with time. Furthermore, a comparison of the microbial community dynamics to neutral model parameters showed a better fit to neutrality in those on ETI. Thus, the airway microbiome changes we observed in people on therapy may represent more random immigration and emigration dynamics, despite pathogen persistence. This increased and more neutral immigration may be sourced from the upper airway, a phenomenon characterizing the airway microbiome of healthy subjects without chronic disease [50–52]. A major question of the future of CF lung infections is will the lung microbiome reach a new steady state while on ETI or if will it constantly improve, with pathogens progressively eliminated with time. If a new steady state is reached, determining its structure and function and effect on airway inflammation will be of paramount importance. Modeling the immigration rate over time predicted a negative trend, possibly reflecting the re-establishment of a new configuration, but further work is needed to determine if the microbiome and metabolome of CF airways have reached a new steady state with the broad administration of ETI.

This study has several potential confounders and limitations that should be accounted for in data interpretation. Firstly, the longitudinal nature of the sampling approach was not uniform, as some subjects provided more samples than others and the sampling starting points were not at a consistent time since ETI began. This is due to the opportunistic and non-interventional nature of the sampling approach and the challenges of collection during the COVID-19 pandemic. Another limitation is that samples were only collected from subjects who could produce sputum at home, which may not represent the ETI response of the complete CF population. The ability of an individual to expectorate a sample is also likely to vary, even in subjects considered ‘sputum producers’ on ETI. Regardless of these limitations, the in-home opportunistic sampling approach provided a unique view into the sputum microbiome and metabolome dynamics in individuals taking ETI and enabled the collection of samples that are more difficult to produce spontaneously in the clinical environment. Another potential confounder is the administration of antibiotics during the collection period, which may have affected the microbiome data, as it has been shown that antibiotics can induce complex changes in the CF lung microbiome [53]. Comparing the presence of antibiotics directly in the sputum from the metabolome data with the microbiome diversity showed that azithromycin may have impacted microbial profiles, but other drugs did not have associations and not all subjects were on the same antibiotics, so this impact is likely minimal overall. Another potential confounder is that the data generation methods from the initial longitudinal collections prior to ETI approval [18] had somewhat different methodologies, including a different DNA extraction protocol and the use of a different mass spectrometer. Differences in DNA extraction kits may have affected microbiome profiles in this study and the different mass spectrometers used between the two data sets could have introduced variations to the metabolite profiles. However, these confounders have been mostly minimized here because the data comparisons were only done after calculating variation within a subject themself through time, which would somewhat normalize any across-group variability affected by different methodologies. Despite these important confounders, this study provided a unique look into the high-resolution microbiome and metabolome dynamics of pwCF on ETI, showing that infection persists and has become more variable and neutral. Further study of changes in the airway microbiology and biochemistry of pwCF taking highly effective modulators will reveal the future infection landscape of this rapidly improving chronic lung disease.

### Supplementary Information

**Additional file 1.** Supplementary methods, tables, figures.

## Data Availability

The microbiome data is currently available at Qiita at: https://qiita.ucsd.edu/analysis/description/53908/. The metabolome data regarding the subjects on ETI is publicly available at GNPS as: https://gnps.ucsd.edu/ProteoSAFe/status.jsp?task=458123f465e24c55acc01d76be6cd765.

## Abbreviations

Ac: *Achromobacter *sp*.*
AIC: Akaike information criterion
ASV: Amplicon sequence variant
Bc: *Burkholderia cepacia*
BMI: Body mass index
CF: Cystic fibrosis
CFTR: Cystic fibrosis transmembrane conductance regulator
COVID-19: Coronavirus disease 2019
DNA: Deoxyribonucleic acid
EBI: European Bioinformatic Institute
ETI: Elexacaftor/Tezacaftor/Ivacaftor
FDA: U.S. Food and Drug Administration
FEV1: Forced expiratory volume in 1 s
FVC: Forced Vital Capacity
GNPS: Global Natural Products Social
GPLs: Glycerophospholipids
IRB: Institutional Review Board
LL: Log-likelihood
m/z: Mass-to-charge ratio
MS/MS: Tandem mass spectrometry
NHQ: 2-Nonylquinolin-4(1H)-one
Pa: *Pseudomonas aeruginosa*
PCR: Polymerase chain reaction
ppFEV1: Percent predicted Forced expiratory volume in 1 s
pwCF: Person with Cystic Fibrosis
qPCR: Quantitative polymerase chain reaction
qTOF: Quadrupole Time of Flight
RF: Random Forest
RMSE: Residual mean square error
rRNA: Ribosomal ribonucleic acid
Sa: *Staphylococcus aureus*
SD: Standard deviation
Steno: *Stenotrophomonas *sp*.*
Strep: *Streptococcus *sp.
UCSD: University of California in San Diego
