# Supplementary material for: Longitudinal microbial and molecular dynamics in the cystic fibrosis lung after Elexacaftor–Tezacaftor–Ivacaftor therapy
Source: Respir Res. 2023 Dec 16;24:317. doi: 10.1186/s12931-023-02630-z (PMC10725582; doi:10.1186/s12931-023-02630-z)
Supplement: Supplementary file 1 — Additional file 1. Supplementary methods, tables, figures. [file 12931_2023_2630_MOESM1_ESM.docx]

Additional file

**Longitudinal Microbial and Molecular Dynamics in the Cystic Fibrosis Lung after Elexacaftor-Tezacaftor-Ivacaftor therapy**

Christian Martin ^a^, Douglas V. Guzior ^a,b^, Cely T. Gonzalez ^a^, Maxwell Okros ^a^,Jenna Mielke ^c^, Lienwil Padillo ^c^, Gabriel Querido ^c^, Marissa Gil ^c^, Ryan Thomas ^d^, Marc McClelland ^e^, Doug Conrad ^c^, Stefanie Widder ^f#^, Robert A. Quinn ^a∗#^

1. Department of Biochemistry and Molecular Biology, Michigan State University, East Lansing, MI, USA
2. Department of Medicine, University of California San Diego, La Jolla, CA, USA
3. Department of Microbiology and Molecular Genetics, Michigan State University, East Lansing, MI, USA
4. Department of Pediatrics and Human Development, Michigan State University, East Lansing, MI, USA
5. Spectrum Health, Grand Rapids, MI, USA
6. Department of Medicine, Research Division Infection Biology, Medical University of Vienna, Austria

^#^Denotes Co-last author

^*^ Denotes corresponding author: Robert A. Quinn, 603 Wilson Rd. Rm 120, Department of Biochemistry and Molecular Biology, Michigan State University, East Lansing, Michigan, 48824, [quinnrob@msu.edu](mailto:quinnrob@msu.edu)

Supplementary methods.

**MZmine 3 settings**

*Mass detection*

Raw data files: Evaluation not executed.

Scans: MS level (1), Scan definition ()

Scan types (IMS): All scan types

Mass detector: Centroid

Noise level: 200000.0

Detect isotope signals below noise level: false

m/z tolerance: 5.0E-4 m/z or 10.0 ppm

Maximum charge of isotope m/z: 1

*Mass detection*

Raw data files: Evaluation not executed.

Scans: MS level (2), Scan definition ()

Scan types (IMS): All scan types

Mass detector: Centroid

Noise level: 5000.0

Detect isotope signals below noise level: false

m/z tolerance: 5.0E-4 m/z or 10.0 ppm

Maximum charge of isotope m/z: 1

*ADAP Chromatogram Builder*

Raw data files: Evaluation not executed.

Scans: MS level (1),

Min group size in # of scans: 5

Group intensity threshold: 200000.0

Min highest intensity: 200000.0

Scan to scan accuracy (m/z): 0.02 m/z or 5.0 ppm

*Local minimum feature resolver*

Feature lists: Aligned feature list filtered_100_850Da subtracted_1e04

Suffix: resolved

Original feature list: KEEP

MS/MS scan pairing: false

Retention time tolerance: 0.1 minutes

MS1 to MS2 precursor tolerance (m/z): 0.02 m/z or 5.0 ppm

Limit by RT edges: true

Combine MS/MS spectra (TIMS): false

Lock to feature mobility range: false

Minimum merged intensity (absolute, IMS): false (250.0)

Minimum merged intensity (relative, IMS): true (0.01)

Dimension: Retention time

Chromatographic threshold: 0.8

Minimum search range RT/Mobility (absolute): 0.02

Minimum relative height: 0.01

Minimum absolute height: 200000.0

Min ratio of peak top/edge: 2.0

Peak duration range (min/mobility): [0.0..0.2]

Min # of data points: 4

*Group MS2 scans with features*

Feature lists:

Retention time tolerance: 0.1 minutes

MS1 to MS2 precursor tolerance (m/z): 0.02 m/z or 5.0 ppm

Limit by RT edges: true

Combine MS/MS spectra (TIMS): false

Lock to feature mobility range: false

Minimum merged intensity (absolute, IMS): true (200000.0)

Minimum merged intensity (relative, IMS): true (0.02)

*13C isotope filter (formerly: isotope grouper)*

Name suffix: deisotoped

m/z tolerance: 0.02 m/z or 5.0 ppm

Retention time tolerance: 0.2 minutes

Mobility tolerance: false (null)

Monotonic shape: false

Maximum charge: 5

Representative isotope: Most intense

Never remove feature with MS2: false

Original feature list: KEEP

*Joining aligner*

Feature list name: Aligned feature list

m/z tolerance: 0.02 m/z or 5.0 ppm

Weight for m/z: 75.0

Retention time tolerance: 0.2 minutes

Weight for RT: 25.0

Mobility tolerance: false (null)

Mobility weight: 1.0

Require same charge state: false

Require same ID: false

Compare isotope pattern: false

Isotope m/z tolerance: 0.001 m/z or 5.0 ppm

Minimum absolute intensity: 10000.0

Minimum score: 0.9

Compare spectra similarity: false

Spectral m/z tolerance: 0.001 m/z or 10.0 ppm

MS level: 2

Compare spectra similarity: Weighted dot-product cosine

Weights: MassBank (mz^2 * I^0.5)

Minimum cos similarity: 0.7

Handle unmatched signals: KEEP ALL AND MATCH TO ZERO

Original feature list: KEEP

*Feature list blank subtraction*

Aligned feature list:

Blank/Control raw data files: Evaluation not executed.

Minimum # of detection in blanks: 20

Fold change increase: true (10.0)

Suffix: subtracted_1e04

*Filtering feature list rows*

Feature lists: Aligned feature list filtered_100_850Da subtracted_1e04

Minimum features in a row (abs or %): true (2.0)

Minimum features in an isotope pattern: true (3)

Validate 13C isotope pattern: true

m/z tolerance: 5.0E-4 m/z or 10.0 ppm

Max charge: 1

Estimate minimum carbon: true

Remove if 13C: true

Remove redundant isotope rows: false

m/z: true ([100.0..850.0])

Retention time: false ([2.0..10.0])

features duration range: false ([0.0..10.0])

Chromatographic FWHM: false ([0.0..1.0])

Charge: false ([1..2])

Kendrick mass defect: false

Kendrick mass defect: [0.0..1.0]

Kendrick mass base:

Shift: 0.0

Charge: 1

Divisor: 1

Use Remainder of Kendrick mass: false

Parameter: No parameters defined

Only identified?: false

Text in identity: false ()

Text in comment: false ()

Keep or remove rows: Keep rows that match all criteria

Feature with MS2 scan: true

Never remove feature with MS2: true

Reset the feature number ID: true

Mass defect: false (null)

Original feature list: KEEP

**Molecular networking settings**

A molecular network was created with the Feature-Based Molecular Networking (FBMN) workflow(1). The mass spectrometry data were first processed with MZmine3 and the results were exported to GNPS for FBMN analysis. MS/MS spectra were window filtered by choosing only the top 4 fragment ions in the +/- 0.02 Da window throughout the spectrum. The precursor ion mass tolerance was set to 0.02 Da and the MS/MS fragment ion tolerance to 0.02 Da. A molecular network was then created where edges were filtered to have a cosine score above 0.65 and more than 4 matched peaks. Further, edges between two nodes were kept in the network if and only if each of the nodes appeared in each other’s respective top 10 most similar nodes. Finally, the maximum size of a molecular family was set to 100, and the lowest scoring edges were removed from molecular families until the molecular family size was below this threshold. The spectra in the network were then searched against GNPS spectral libraries (2). The library spectra were filtered in the same manner as the input data. All matches kept between network spectra and library spectra were required to have a score above 0.65 and at least 4 matched peaks. The molecular networks were visualized using Cytoscape software (3).

Supplementary tables

| Subject ID | Inhaled Antibiotic | Azithromycin |
| --- | --- | --- |
| 010 | Cayston | No |
| 011 | Cayston, Tobi | Yes |
| 012 | Cayston | Yes |
| 013 | Cayston | No |
| 014 | None | Yes |
| 015 | Cayston | No |
| 016 | None | No |

Table S1. Antibiotic treatment recorded on every single subject on ETI.

| Subject ID | Variance Explained (Microbiome) | Variance Explained (Metabolome) |
| --- | --- | --- |
| 010 | 38.88% | 3.38% |
| 011 | 55.95% | 79.95% |
| 012 | 35.08% | 24.01% |
| 013 | 28.65% | 28.40% |
| 014 | -6.55% | 26.04% |
| 015 | 20.22% | 28.97% |
| 016 | 38.00% | 4.46% |

Table S2. Random forest variable explained scores for both, the metabolome and microbiome when evaluated their association with the time in which samples were taken after treatment.

Supplementary figures


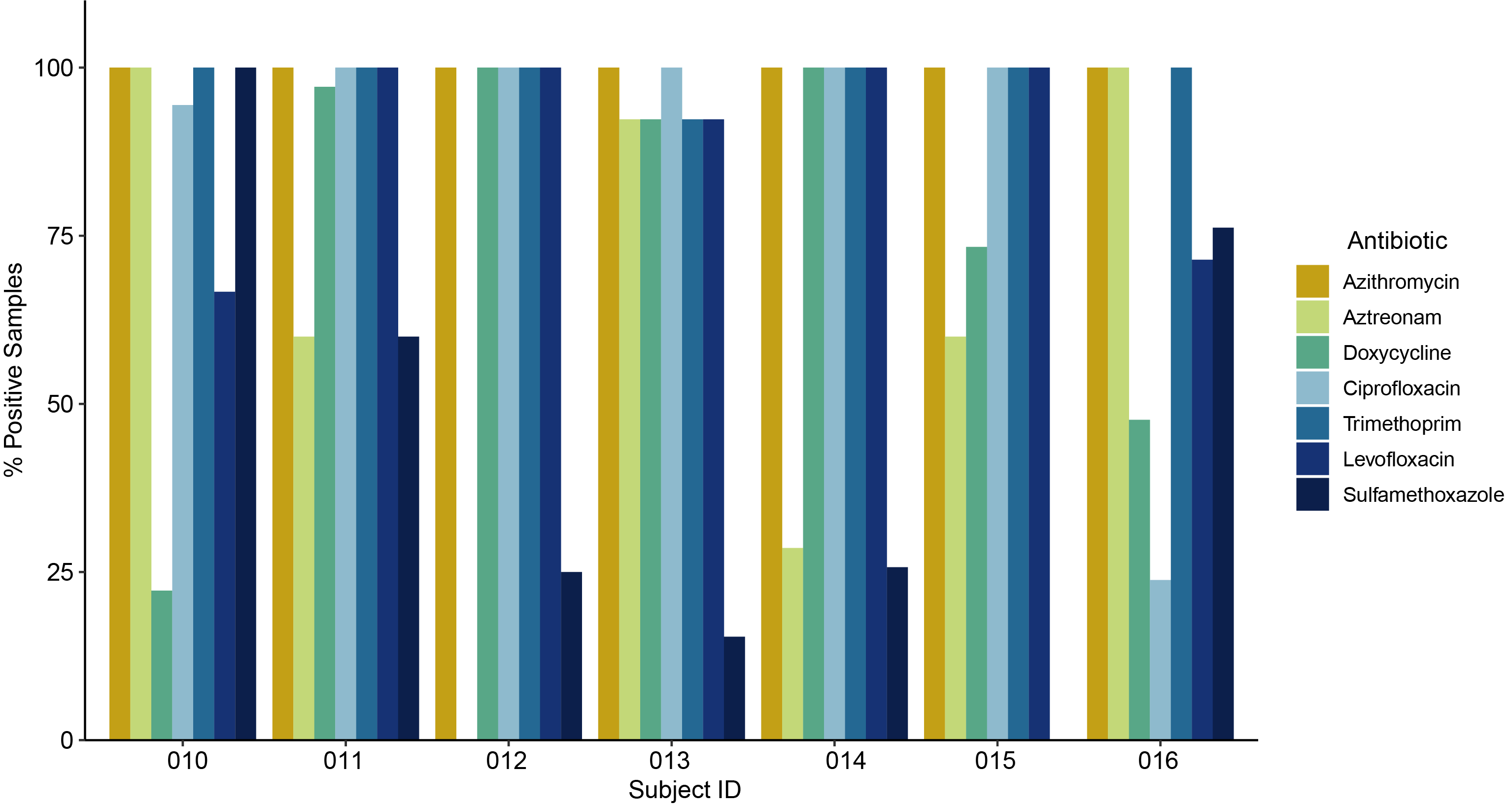


Figure S1. Proportion of sputum samples from subjects of ETI where antibiotics were detected through LC-MS/MS. Azithromycin was found among all patients while aztreonam was detected in all patients except subject 012.


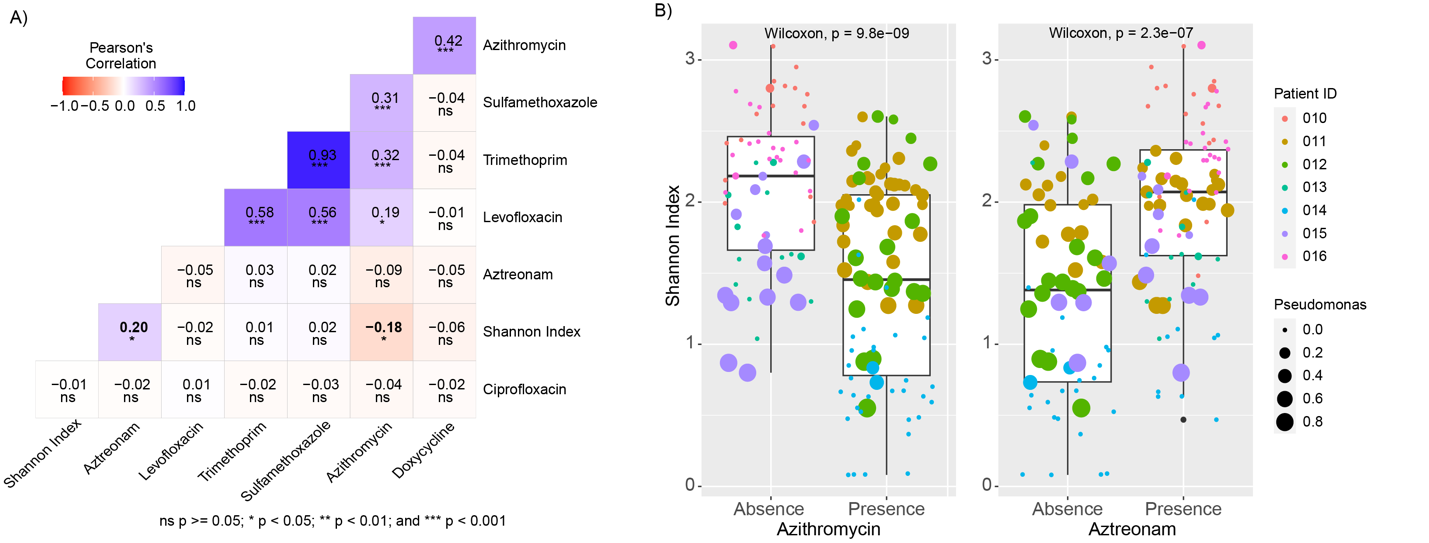


Figure S2. A) Pearson’s correlation heatmap for the antibiotics detected in sputum samples and the Shannon index (alpha-diversity variation). Azithromycin and Aztreonam significantly increased and decreased respectively when correlated with the Shannon index. B) Boxplots of the Shannon index variation when azithromycin and aztreonam were annotated and not annotated by GNPS. Jitter’s colors correspond to the subject id while the size correspond to the relative abundance of *Pseudomonas* on each sample. Wilcoxon test was performed for determine the significance based on the antibiotic’s annotations.


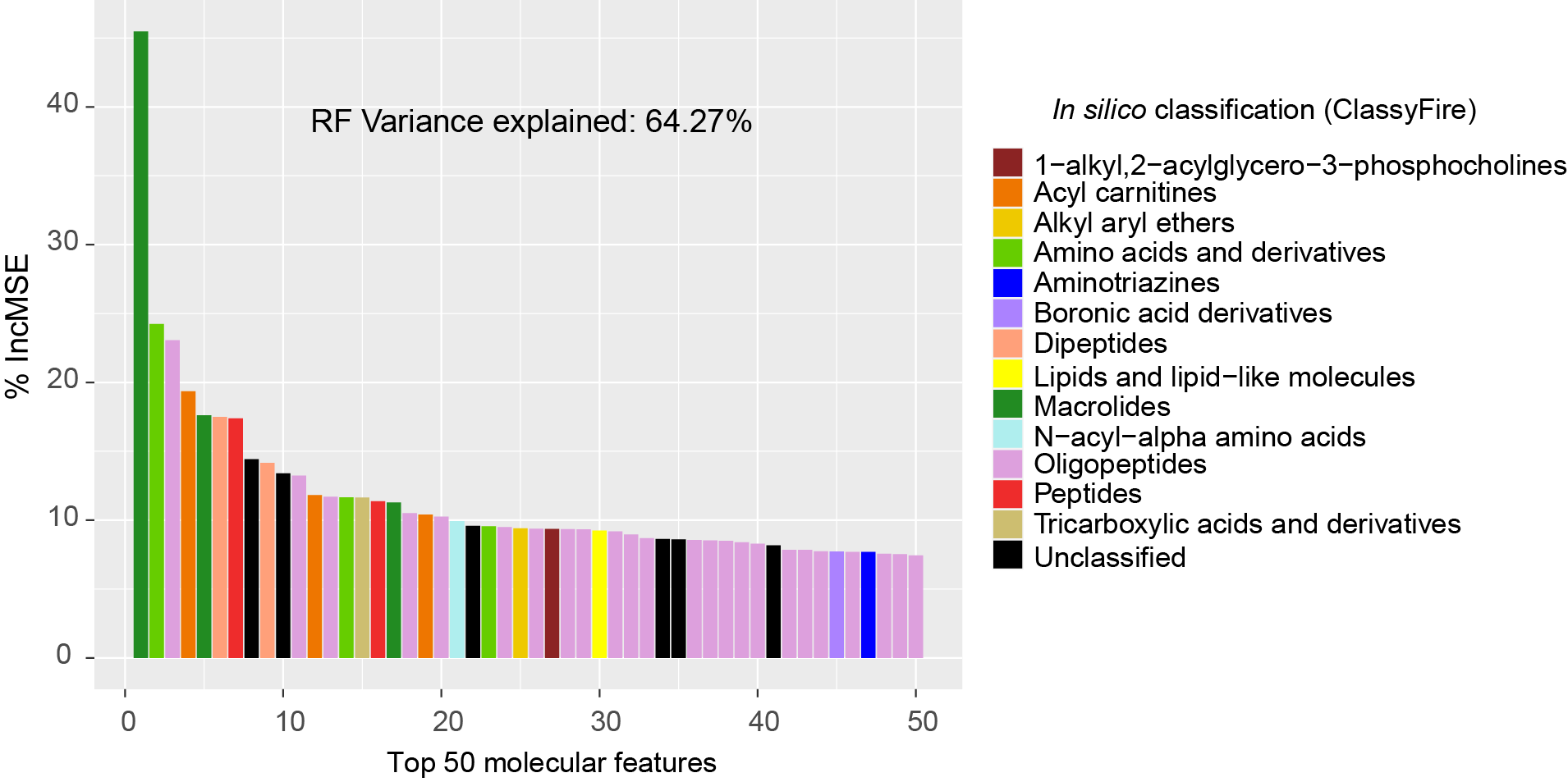


Figure S3. Top 50 molecular features obtained after random forest regression analysis of the metabolome of sputum from pwCF on ETI and the time since the overall subject started on the treatment. Colors represent the *in silico* molecular classification generated through CANOPUS in SIRIUS (version 5.7).


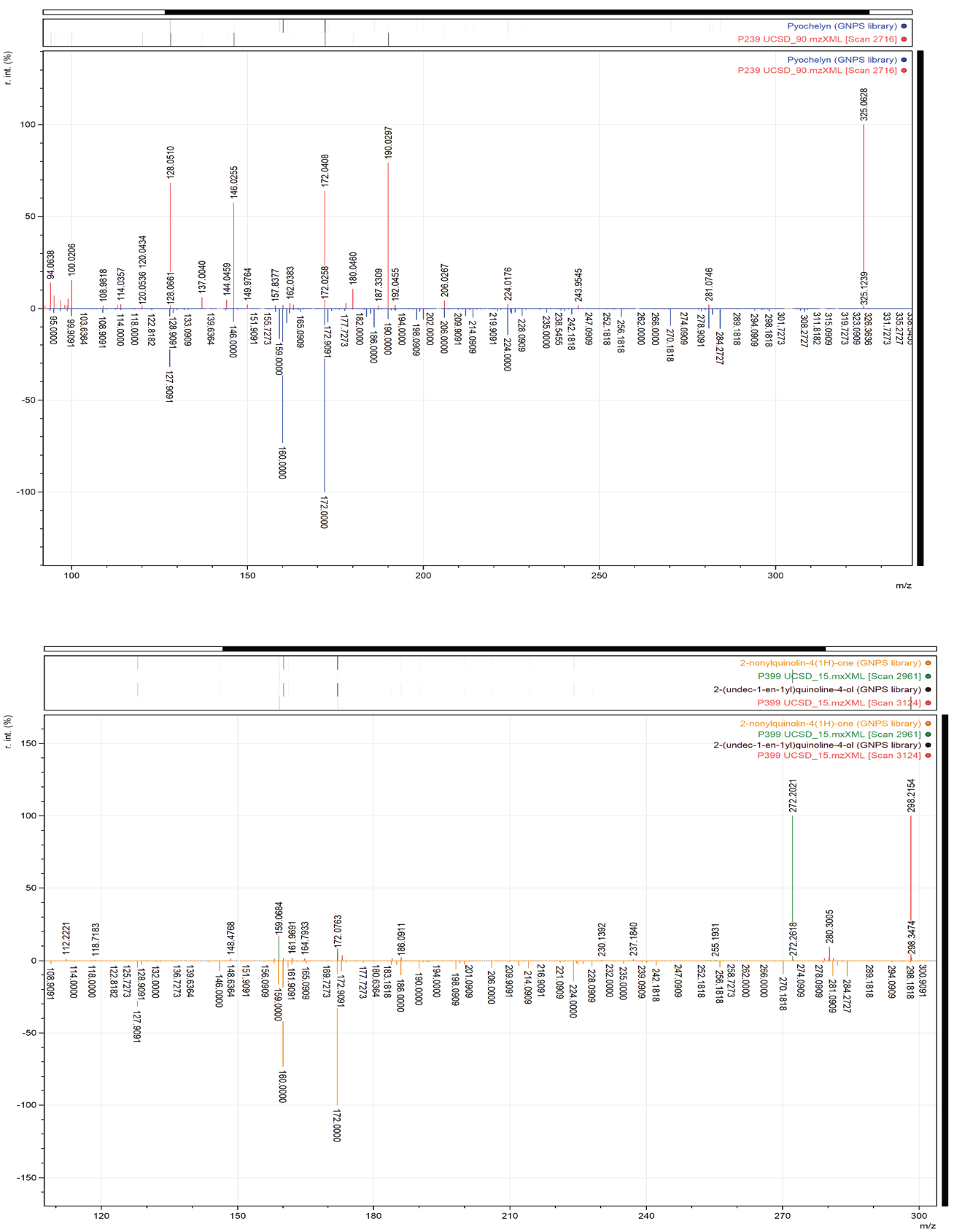


Figure S4. MS/MS patterns through GNPS mass spectral libraries. Pyochelin ([M+H^+^], *m*/*z* = 325.0625), 2-nonylquinolin-4(1*H*)-one ([M+H^+^], *m*/*z* = 272.1995) and 2-(undec-1-en-1yl)quinoline-4-ol ([M+H^+^], *m*/*z* = 298.2165) are displayed.

Supplementary references

1. Nothias LF, Petras D, Schmid R, Dührkop K, Rainer J, Sarvepalli A, et al. Feature-based molecular networking in the GNPS analysis environment. Nat Methods. 2020 Sep;17(9):905–8.

2. Reproducible molecular networking of untargeted mass spectrometry data using GNPS | Nature Protocols [Internet]. [cited 2023 May 8]. Available from: https://www.nature.com/articles/s41596-020-0317-5

3. Cytoscape: A Software Environment for Integrated Models of Biomolecular Interaction Networks [Internet]. [cited 2023 May 8]. Available from: https://genome.cshlp.org/content/13/11/2498
